# Homogeneous and heterogeneous molecular catalysts for electrochemical reduction of carbon dioxide

**DOI:** 10.1039/d0ra07973a

**Published:** 2020-10-15

**Authors:** Maryam Abdinejad, M. Nur Hossain, Heinz-Bernhard Kraatz

**Affiliations:** Department of Physical and Environmental Sciences, University of Toronto Scarborough 1265 Military Trail Toronto ON M1C 1A4 Canada bernie.kraatz@utoronto.ca

## Abstract

Carbon dioxide (CO_2_) is a greenhouse gas whose presence in the atmosphere significantly contributes to climate change. Developing sustainable, cost-effective pathways to convert CO_2_ into higher value chemicals is essential to curb its atmospheric presence. Electrochemical CO_2_ reduction to value-added chemicals using molecular catalysis currently attracts a lot of attention, since it provides an efficient and promising way to increase CO_2_ utilization. Introducing amino groups as substituents to molecular catalysts is a promising approach towards improving capture and reduction of CO_2_. This review explores recently developed state-of-the-art molecular catalysts with a focus on heterogeneous and homogeneous amine molecular catalysts for electroreduction of CO_2_. The relationship between the structural properties of the molecular catalysts and CO_2_ electroreduction will be highlighted in this review. We will also discuss recent advances in the heterogeneous field by examining different immobilization techniques and their relation with molecular structure and conductive effects.

## Abstract 2

Electroreduction of CO_2_ to CO using molecular catalysis.

## Graphical abstract

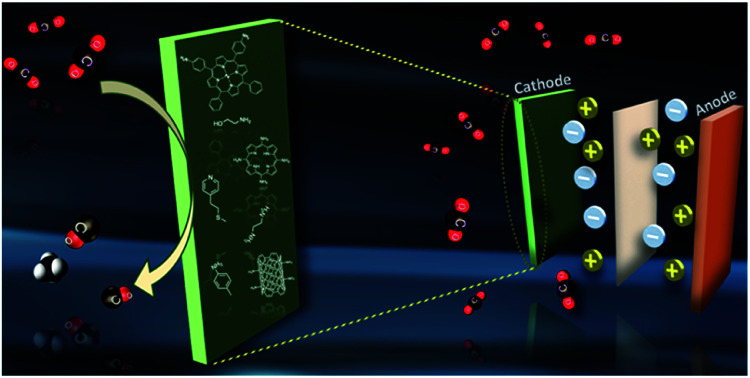

## 1. Introduction

Carbon dioxide (CO_2_), as a greenhouse gas, is a significant contributor to climate change. The global average atmospheric CO_2_ level in 2019 was 409.8 ppm, much higher than the previous highest concentration of 300 ppm, with levels projected to keep increasing unless immediate measures are taken.^1,2^ These emission levels have raised serious environmental concerns and have translated to noticeable, aberrant meteorological changes.

Recent strategies that convert CO_2_ into value-added materials using photochemically^3^ or electrochemically^4–6^ powered reduction reactions have shown promise in recent years. However, this task is challenging due to the high energy required (750 kJ mol^−1^) to break the C=O bond^7,8^ and the molecule's stable linear geometry, which makes CO_2_ reduction reactions (CO_2_RRs) sluggish and challenging.^3,9^ Additionally, the electrocatalytic CO_2_ reduction mechanism is a complex process that involves multiple proton-coupled electron transfer steps and may include several side-reactions and intermediates.^10–14^

The first step of many CO_2_RRs is the one-electron reduction of CO_2_ to a CO_2_˙^−^ radical anion which has a more reactive, bent geometry (Table 1).^15,16^ Although most CO_2_RRs describe two-electron reduction to carbon monoxide (CO) and formaldehyde, products of multi-electron transformations such as methane,^17^ methanol^18^ and ethanol^19^ are highly coveted. Table 1 shows the theoretical potentials required to form various multi-electron reductions. Although the theoretical potentials required to form the target products shown in Table 1 appear relatively low, because the products formed are often either thermodynamically similar or more stable than CO_2_, more negative potentials are required for practical applications to obtain reasonable reaction rates.^9^ In order to facilitate CO_2_RR, the use of catalysts is essential and serves several purposes including lowering activation energy barriers, improving reaction rates, and increasing product selectivity.^20–23^

**Table 1:** Electrochemical potentials of possible CO_2_RR in aqueous solutions^24^

CO_2_ reduction half-reactions	Electrode potentials (V *vs.* NHE) at pH = 7
CO_2_ + e^−^ → CO_2_˙^−^	−1.90
CO_2_ + 2H^+^ + 2e^−^ → HCO_2_H	−0.61
CO_2_ + 2H^+^ + 2e^−^ → CO + H_2_O	−0.53
CO_2_ + 4H^+^ + 4e^−^ → HCHO + H_2_O	−0.48
CO_2_ + 6H^+^ + 6e^−^ → CH_3_OH + H_2_O	−0.38
CO_2_ + 8H^+^ + 8e^−^ → CH_4_ + 2H_2_O	−0.24

Electroreduction of CO_2_RR can be completed using either homogeneous or heterogeneous catalysts. Although homogeneous catalysis have shown high selectivity, with near product unity for the production of CO and other reduction products,^18,25–29^ these systems are dependent on the solubility constraints of the catalysts and are limited by low current densities and instability.^30^ On the other hand, heterogeneous electrocatalysts minimize the electrode and catalyst distance, allowing for more efficient processes and higher current densities at the expense of product selectivity.^31–33^ In either case, although a variety of electrocatalysts have been introduced for CO_2_RR in recent decades,^34–36^ the performance of these systems has yet to reach a level where they can be successfully implemented industrially.^37,38^

Recent advancements have found success through incorporating a combination of molecular catalysts and heterogeneous immobilization strategies.^39–41^ Different molecular approaches such as metal organic frameworks (MOFs), covalent organic frameworks (COFs), and metal-free catalysts have tried to address this issue.^31,42–45^ It has been shown that applying organic compounds, such as thiols,^46,47^ polypyrrole,^48^ N-heterocyclic carbenes (NHCs),^49^ and N-substituted pyridines,^17,50,51^ can reduce CO_2_ to desirable materials such CO, HCOO^−^ and COOH.

**Scheme 1:**
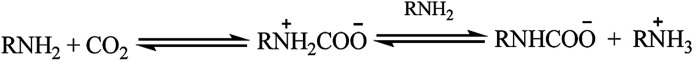
Carbamate formation using primary and secondary amines.

The amino functional group in particular has proven to be effective at selectively capturing CO_2_ from a mixture of gases. This ability is especially pronounced in primary or secondary amines such as monoethanolamine (MEA), diethanolamine (DEA) and decylamine (DCA).^52–57^ In these reactions, the amino groups initially capture CO_2_ to form a zwitterionic species that can react with another amino equivalent to form a carbamate salt (Scheme 1).^58,59^

This review will start with a general introduction to the electrochemical reduction of CO_2_ and the metrics that are used to quantify catalyst efficiency and continue with a summary of the recent developments of amine molecular catalysts in both homogeneous and heterogeneous environments. In general, this report suggests that structural tuning of organic compounds followed by either covalent or non-covalent immobilization onto various conductive surfaces (*i.e.*, graphite, Au, Ag, Pd, and Cu), results in high performing systems.

## 2. Electroreduction of carbon dioxide

Electrochemical capture and reduction of CO_2_ ^60^ has received extensive interest in the last decade because of the: (1) controllable nature of the technique (*e.g.* potential and temperature); (2) flexibility between organic and aqueous media; (3) relative scalability of bench-side reaction setups to industrial application.^61^

**Fig. 1:**
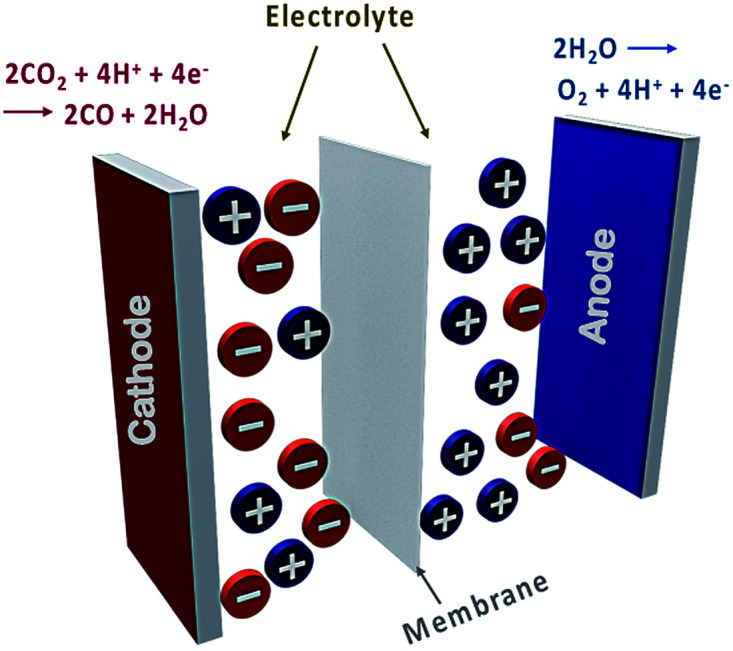
A typical electrochemical CO_2_ reduction reaction cell. The oxidation and reduction occur at anode and cathode, respectively. The membrane separates the compartments. The electrolyte includes positive and negative species that assist charge and CO_2_ transport.

Typical electrochemical cells consist of a cathode, anode, electrolyte and a membrane (Fig. 1). CO_2_RR occurs at the cathode, while reciprocal oxidation or oxygen evolution reactions (OERs) occur at the anode. The cathode and the anode are separated by a membrane which maintains charge balance and separates the respective redox products. The electrolyte carries the charge between the electrodes and delivers dissolved CO_2_ to the catalytically active surface.

## 3. Quantifying catalytic performance

Several factors are used to quantify catalytic performance. Selectivity is measured by the faradaic efficiency (FE), and the catalyst activity is determined by the current density (*j*) as a function of the electrode area. The current density can be used to describe either the total current density of all reduced products or the partial current density of one particular product. In the context of CO_2_RR, current density can be used to describe the rate of reaction. The robustness of the catalyst is calculated with the turnover number (TON) which is determined by dividing the mole of reduced product with the mole of catalyst. The turnover frequency (TOF, s^−1^) is defined as the mole of reduction product divided by the mole of active catalysts per unit of time.

## 4. Molecular electrocatalysis for CO_2_RR

Using electrochemical techniques to reduce captured CO_2_ with small molecules is a promising strategy to produce valuable materials.^62^ This has been demonstrated previously using amino and pyridine-substituted compounds for electrochemical CO_2_RR in both homogeneous and heterogeneous media.^18,63–69^

Various catalysts have been developed as both homogeneous molecular catalysts^16,18,70–77^ and heterogeneous solid-state catalysts,^22,40,78^ such as metal alloys,^79,80^ non-metal catalysts^81^ and single atoms.^82^ The identity of the metal electrodes have also been shown to play a role in the product distribution.^12,83–93^ In this section, recent developments in molecular electrocatalysts for CO_2_ reduction will be discussed with respect to their molecular structure, nano-structuring immobilization and electrode surface modification. The study of the following molecular catalysts highlights the importance of molecular design, electronic factors, and ligand structure in successful experimental design.

### 4.1. Homogeneous amine molecular catalysts and electrochemical CO_2_RR

**Fig. 2:**
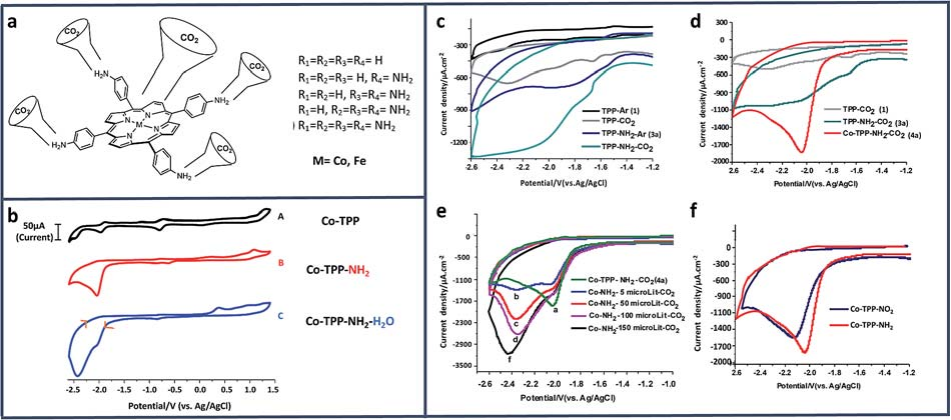
(a) Schematic of metallated amino porphyrins; cyclic voltammograms (CV) of 0.01 mM of (b) Co-TPP, Co-TPP–NH_2_, and Co-TPP–NH_2_ with 5% H_2_O; under CO_2_; (c) comparison of TPP and TPP–NH_2_ under Ar and CO_2_; (d) comparison of TPP, TPP–NH_2_, and Co-TPP–NH_2_ under Ar and CO_2_; (e) Co-TPP–NH_2_ in 0.1 M NBu_4_PF_6_ in DMF solutions at a scan rate of 100 mVs^−1^ in: (a) (no water), (b) 5 μL (C) 50 μL (d) 100 (e) 150 μL water. (f) Comparison of Co-TPP–NO_2_ and Co-TPP–NH_2_ under Ar and CO_2_ in 0.1 M NBu_4_PF_6_ and DMF solutions. Conditions: scan rate, 100 mV s^−1^; working electrode, glassy carbon; reference electrode, Ag/AgCl; counter electrode, platinum.^18^ Copyright (2019) American Chemical Society.

Homogeneous studies of amine-based molecular electrocatalysts have been identified their utility for CO_2_RR. Using *meso*-substituted amino groups on metallated porphyrins, we were able to achieve selective reduction of CO_2_ to CO and methanol (Fig. 2a).^18^ Comparing the cyclic voltammograms of Co-TPP and Co-TPP–NH_2_ in the presence of CO_2_ clearly highlights the importance of the amino group and its role in reducing CO_2_ (Fig. 2b). The influential presence of the cobalt center in CO_2_RR, can be seen in Fig. 2d. In this project, H_2_O was used as an extra proton source to facilitate the C–O bond cleavage (Fig. 2e). To further understand the electroactivity of the amino group, a comparison with nitro porphyrins (TPP–NO_2_) shows a slightly better performance of the amino group (Fig. 2f).

**Fig. 3:**
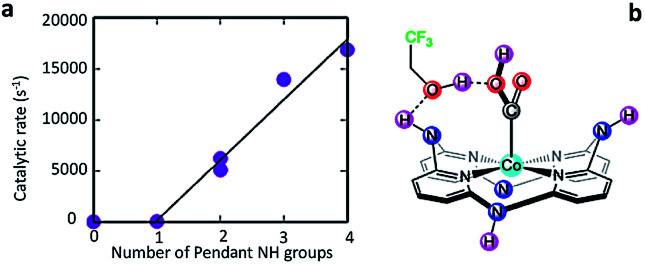
(a) Experimental catalytic rate constants, *k*_obs_ (s^−1^), as a function of the number of pendant secondary amines using 1.5 M TFE under CO_2_ saturation at a scan rate of 100 mV s^−1^. (b) Schematic of pendant hydrogen-bond donors in cobalt catalysts independently enhance CO_2_ electroreduction.^95^ Copyright (2018) American Chemical Society.

Chapovetsky *et al.*^94^ also reported a cobalt aminopyridine macrocycle with amine substituents selectively reducing CO_2_ to CO. From electrochemical experiments, they found that the catalytic activity is strongly dependent on the number of secondary amines (Fig. 3).^95^ Subsequent studies showed how those amine groups could act as hydrogen bond donors to enhance catalytic performance.

The identity of the electrode used has been found to have a large influence on the catalytic activity of homogeneous amine solutions, with different electrodes such as glassy carbon, copper, and silver each eliciting their own distinctive response.^18,46,55,63,96–101^ Lue *et al.*^55^ reported a systematic study on electrochemical CO_2_ reduction with 30% (w/w) MEA on, Sn, Pb, Pd, Ag, Cu and Zn metal electrodes. Schmitt *et al.*^100^ used *in situ* surface-enhanced Raman spectroscopy to study 3,5-diamino-1,2,4-triazole (DAT) exposed-Ag electrodes, finding that the amine treated electrode increased FE_CO_ due to a weakening of the CO bonding strength.

**Fig. 4:**
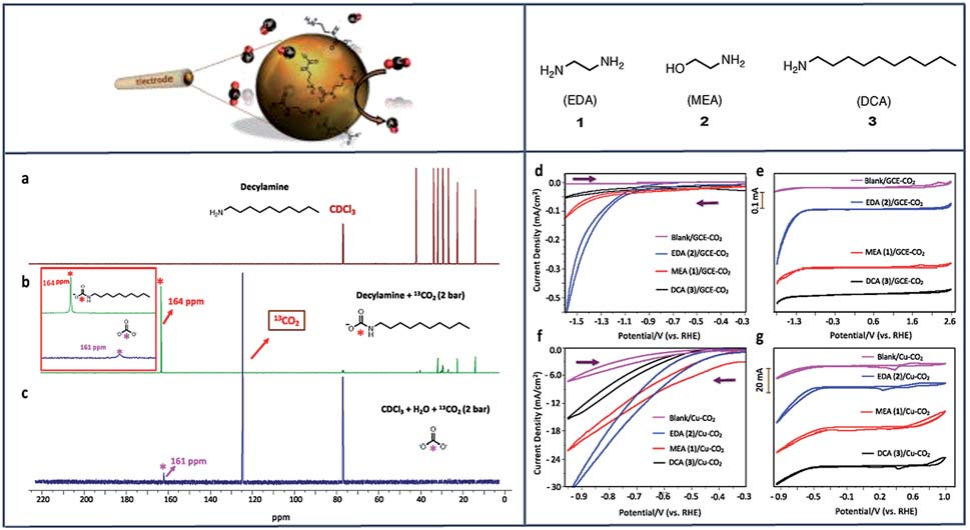
^13^C NMR spectra of DCA (a), DCA–^13^CO_2_ (b) and H_2_O–^13^CO_2_ (c) in CDCl_3_. Cyclic voltammograms (CV) of: (d) 1–3 under CO_2_ with GCE; (e) compounds 1–3 under CO_2_ with GCE stacked; (f) 1–3 under CO_2_ with Cu electrode; (g) 1–3 under CO_2_ with Cu electrode stacked; 0.1 mM concentration in 0.1 M NaClO_4_ solution. Conditions: scan rate, 100 mVs^−1^; working electrode, glassy carbon/copper; reference electrode, Ag/AgCl; counter electrode, platinum.^57^ Copyright (2020) American Chemical Society.

Many studies of copper (Cu) electrodes have characterized their ability to reduce CO_2_ to multi-carbon products,^14,33,55,87,102–110^ whereas when exposed to molecular catalysts it is more common to see CO_2_ selectively converted to CO,^57^ formate,^111^ and formic acid.^13,104^ We have also investigated the ability of primary amines to selectively reduce CO_2_ to CO using Cu electrodes (Fig. 4).^57^ In these studies, ethylenediamine (EDA) proved to be the most effective absorbent for CO_2_ capture and subsequent reduction to CO among MEA and decylamine (DCA), with a current density of −18 mA cm^−2^, TON of 252 and a FE of 58% at −0.78 V *vs.* RHE. Compared to glassy carbon electrodes, the cathodic current was dramatically enhanced when Cu was used as a working electrode (Fig. 4f and g).

Our recent studies on the electrochemical reduction of CO_2_ in various fractions of MEA solutions at smooth and nanodendrite (ND) Cu, Ag and Au showed that a 0.05 M fraction of MEA exhibited the highest catalytic activity for each surface.^112^ CO_2_ electroreduction to HCOO^−^. The ND electrodes exhibited much higher current efficiencies for CO_2_ to HCOO^−^ conversion compared to the smooth metal electrodes, revealing the critical role of surface morphology in enhancing catalytic activity.

### 4.2. Heterogeneous amine molecular catalysts and electrochemical CO_2_RR

Heterogeneous electrocatalysts have benefits over homogeneous electrocatalysts for CO_2_RR application due to the catalytically active site being either located directly on the electrode surface or the electrode itself. As a result, catalytic loading concentrations can be lower. Molecular catalysts can be attached to solid, conductive surfaces *via* covalent/non-covalent immobilization techniques^29,113,114^ or using polymers and metal–organic frameworks.^115–117^ This strategy offers higher stability and catalytic efficiency^56^ with a greater potential of reaching the necessary current densities for industrial implementation.^118^ Due to its simple preparations, one of the most popular immobilization techniques involves depositing conjugated organic ligands onto carbon surfaces which are stabilized by the non-covalent π–π interactions between the catalyst and solid surface.^17,56,119^ The molecular catalysts can be also deposited on electrode surfaces through covalent bond.^120,121^

Previous reports on CO_2_RR selectivity involved either the use of a metal electrode surface, where the electron-transfer efficiency was largely dependent on the material's conductivity, or the incorporation of small inactive molecules^39^ on the surface of the metal electrode to maximize interaction between the electrode and the molecular catalysts.^56,61,122–125^ An example of this are electrografting techniques which produce a direct chemical bond between the catalyst and a solid substrate.^98,126^ The direct connections that arise from these methods are believed to be the primary factor in increasing the reaction rate of CO_2_RR relative to hydrogen evolution reactions (HERs) and lowering overpotentials.^125,127–131^ Using this technique, immobilization of terpyridine onto glassy carbon electrodes has been previously reported.^66^

**Scheme 2:**
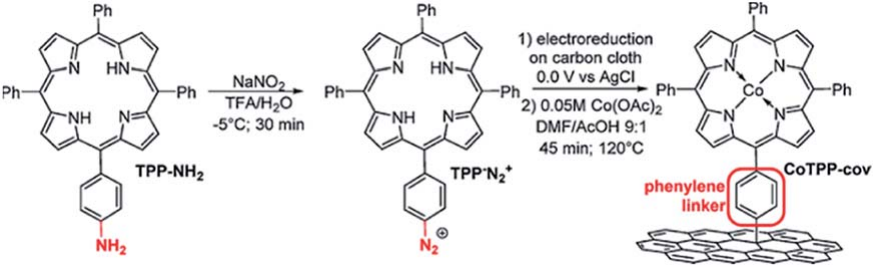
Preparation of covalently immobilized Co tetraphenylporphyrin (CoTPP-cov).^121^ Copyright (2019) Elsevier.

**Fig. 5:**
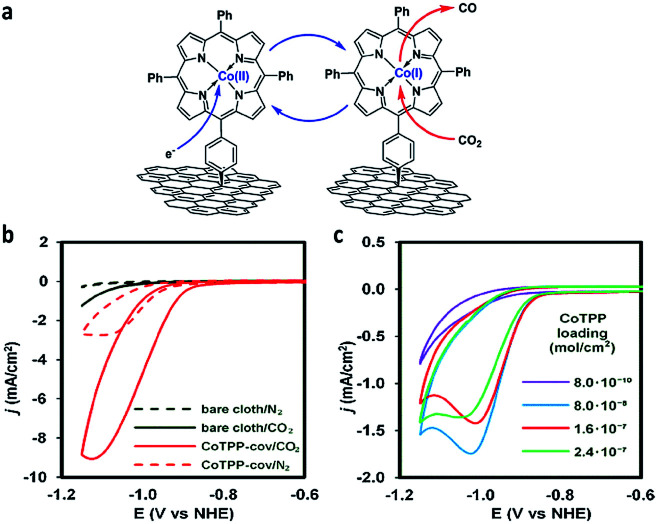
(a) Preparation of covalently immobilised Co tetraphenylporphyrin (CoTPP-cov); (b) CVs of CoTPP-cov in N_2_- and CO_2_-purged aqueous electrolyte, CVs of bare carbon cloth are shown for clarity; (c) CV traces of CoTPP-noncov with the variable amount of noncovalently immobilized CoTPP in CO_2_-saturated solution. Conditions: electrolyte: 0.5 M KHCO_3_ in all cases, potential scan rate is 100 mV s^−1^.^121^ Copyright (2019) Elsevier.

Marianov *et al.*^121^ have also successfully electrografted amino porphyrins *via* electro reduction of diazonium salt onto glassy carbon (Scheme 2). By introducing a conjugated linker between the porphyrin and the electrode, they proved that the Co^I^/Co^II^ redox couple facilitates the CO_2_ electroreduction process (Fig. 5a). With the covalently linked catalyst an increase to the current density (4.7 mA cm^−2^) was seen, compared to the unlinked catalysts (1.4 mA cm^−2^) (Fig. 5b). In addition to the covalent linkage facilitating electrode-to-catalyst charge transfer, the current density was also observed to be dependent on the catalyst loading concentration and the total active surface area (Fig. 5b and c).

**Fig. 6:**
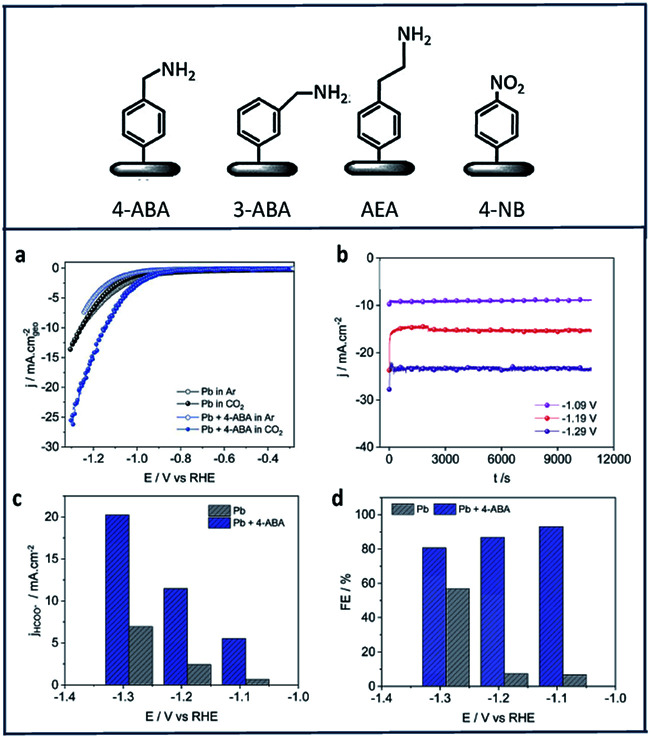
The four different amines used in this work to modify the Pb electrodes: (a) CV of Pb and Pb + 4-ABA; (b) chronoamperograms recorded in CO_2_-saturated 1 M HKCO_3_ solution for Pb + 4-ABA electrode (6.3 × 10^−7^ mol cm^−2^); comparison of (c) current density and (d) faradic efficiency at different potentials for = Pb + 4-ABA and bare Pb electrodes.^97^ Copyright (2019) Royal Society of Chemistry.

Zouaoui *et al.*^97^ investigated the electrocatalytic activity of amine derivatives deposited onto Pb surfaces toward electroreduction of CO_2_ to formate. Using diazonium chemistry, 4-aminomethylbenzene (4-ABA), 3-aminomethylbenzene (3-ABA), 4-(2-aminoethyl)benzene (4-AEA) and 4-nitrobenzene (4-NB) were grafted onto Pb electrodes (Fig. 6). The Pb-amine modified electrodes showed enhanced activity and selectivity in all cases (Fig. 6a). Fig. 6b shows chronoamperograms of the 4-ABA modified Pb electrode (6.3 × 10^−7^ mol cm^−2^) in a 1 M HKCO_3_ solution saturated with CO_2_. In this study, 4-ABA reached a maximum current density of −24 mA cm^−2^ at −1.29 V *vs.* RHE, with a FE over 80% (Fig. 6c and d).

**Fig. 7:**
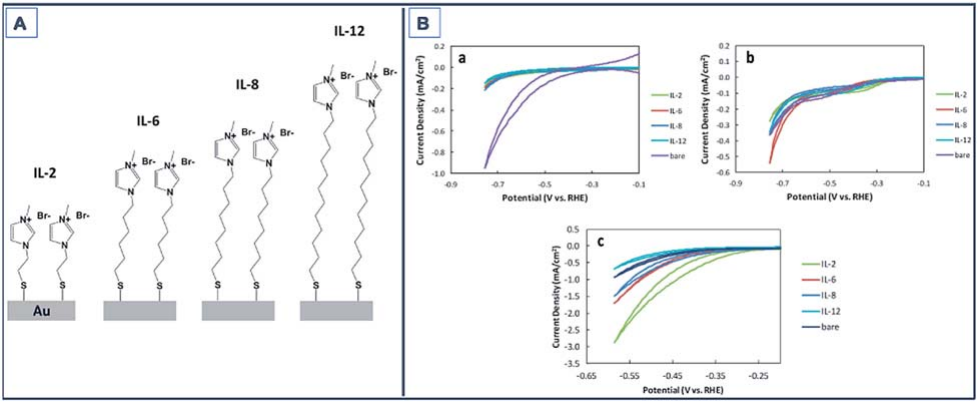
Schematic of Au electrodes with 1-methylimidazolium-terminated SAMs (IL-2, IL-6, IL-8, and IL-12). CV of bare and SAM modified Au electrodes in Na_2_SO_4_ aqueous solution purged with (a) N_2_ and (b) CO_2_. Scan rate: 100 mV s^−1^; (c) CV of bare and SAM-modified Au electrodes in NaHCO_3_ aqueous solution purged with CO_2_. Scan rate: 100 mV s^−1^.^135^ Copyright (2015) Royal Society of Chemistry.

Gold (Au) has been also found to exhibit catalytic activity towards CO_2_RR.^132–134^ Mikoshiba *et al.*^135^ showed that imidazolium ions immobilized on Au electrodes suppress H_2_ generation and accelerate CO_2_RR. In their study, imidazolium salts with small methylene units (IL-2, Fig. 7A) exhibited greater current densities compared to longer chained units with FEs up to 87% (Fig. 7B).

**Fig. 8:**
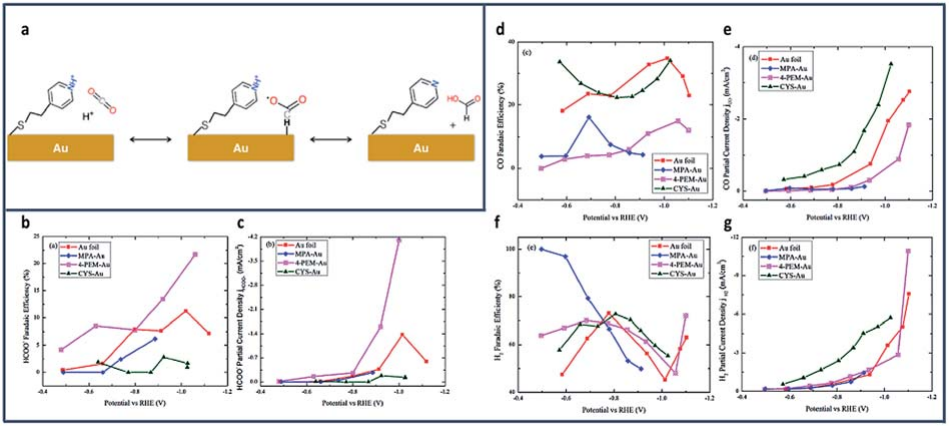
(a) Proposed Mechanism for reduction to formate at PEM-modified Au Electrode; comparison of partial current density and FE for thiolate ligand on polycrystalline Au and pure polycrystalline Au: (b) FE of formate formation (±2.5% at 95% confidence level (CL)), (c) FE of CO formation (±6.2% at 95% CL), (d) FE of H_2_ formation (±25% at 95% CL); (e) partial current density of formate formation; (f) partial current density of CO formation, and (g) partial current density of H_2_ formation.^136^ Copyright (2020) American Chemical Society.

In another study, Au electrodes functionalized with 4-pyridinylethanemercaptan (PEM) thiols showed similar increases in product selectivity and catalytic activity (Fig. 8a).^136^ The proposed mechanism for formate production shows the pyridine H-atom abstracted by reduction of the aqueous solution and adsorbed onto the Au surface (Fig. 8a). HCO_2_ is formed through electrophilic attack of CO_2_ onto the adsorbed proton. The FE of the electroreduction products in this system were observed to be potential dependent. Fig. 8b–g shows the potential-dependent product distribution (formate, CO and H_2_) of functionalized Au and bare Au surfaces.

A 2-fold increase in FE_formate_ and a 3-fold increase in current density were achieved and attributed to enhancement of proton and electron transfers using Au foil (Fig. 8b and c).^137^ This increase in current density is due to the amine's ability to make a complex with CO_2_ near the Au surface.^138^ Cystemine modified electrodes saw a 2-fold increase in both CO and H_2_ production (Fig. 8d–f), while electrodes with 2-mercaptopropionic acid (MPA) ligands reported nearly 100% selectivity for H_2_ (Fig. 8g).

**Fig. 9:**
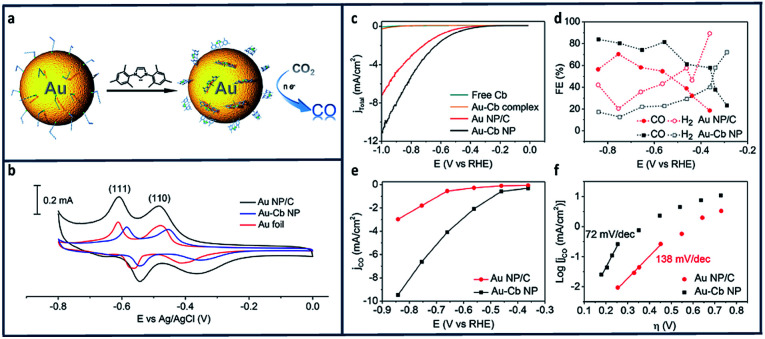
(a) Schematic reduction of CO_2_ using N-heterocyclic (NHC) carbene-functionalized on a gold electrode. (b) Pb-upd profiles of the Au NP/C, Au–Cb NP, and Au–Cb NP was referenced to the geometric area of the Au foil with scan rate of 50 mV s^−1^ (c) LSV scans of Au–Cb NP, Au NP/C, free carbene and molecular Au–Cb complex under CO_2_-saturated 0.1 M KHCO_3_ at pH 6.8. (d) FEs of reduction product formed from Au–Cb NP and Au NP/C. (e) Specific CO current density (based on electrochemically active surface area) plots for Au–Cb NP and Au NP/C. (f) Tafel plots of Au–Cb NP and Au NP/C.^139^ Copyright (2016) American Chemical Society.

In another study, it was found that immobilization of Au nanoparticles using N-heterocyclic carbenes facilitated electron transfer from Au to CO_2_ (Fig. 9a).^139^ The electrochemical reduction of CO_2_ to CO catalysed by a Au-1,3-bis(2,4,6 trimethylphenyl)imidazol-2-ylidene nano particle (Au–Cb NP) was found to be greater than that of bare Au nanoparticles (Au NP). Oleylamine-capped Au NPs (Au–Oa NP) were first loaded onto carbon black to make a Au–Oa NP/C mixture.^140^ The active surface area for Au NP/C and Au-1,3-bis(2,4,6 trimethylphenyl)imidazol-2-ylidene nano particle (Au–Cb NP) electrode were evaluated using Pb underpotential deposition (upd).^141–143^ The current density increased substantially (Fig. 9c and e) and the FE_CO_ increased from 53% to 83% in when the Au nanoparticles were deposited onto CB (Fig. 9d). The kinetics of the CO_2_ reduction were examined using Tafel analysis (Fig. 9f) which shows a decreasing slope from 138 mV dec^−1^ to 72 mV dec^−1^.

Other promising active electrocatalytic systems incorporate Ag metal centers or Ag electrodes.^47,63,144–147^ Compared to Au electrocatalysts, Ag catalyst are cheaper and have comparable activity. Various strategies, such as morphology-nanostructuring have been paired with these electrodes.^148,149^ Hwang and co-workers^100^ prepared three different types of Ag nanoparticles with different surface capping agents. These included oleylamine (OLA), having an amine functional group; oleic acid, having a carboxyl functional group; and dodecanethiol (DDT) with a thiol functional group. They discovered that the amine substituent was highly effective in enhancing the electrochemical reduction of CO_2_ to CO with high selectivity (FE = 94%) at low overpotentials (−0.75 V *vs.* RHE) due to an exceptional suppression of HER.

**Fig. 10:**
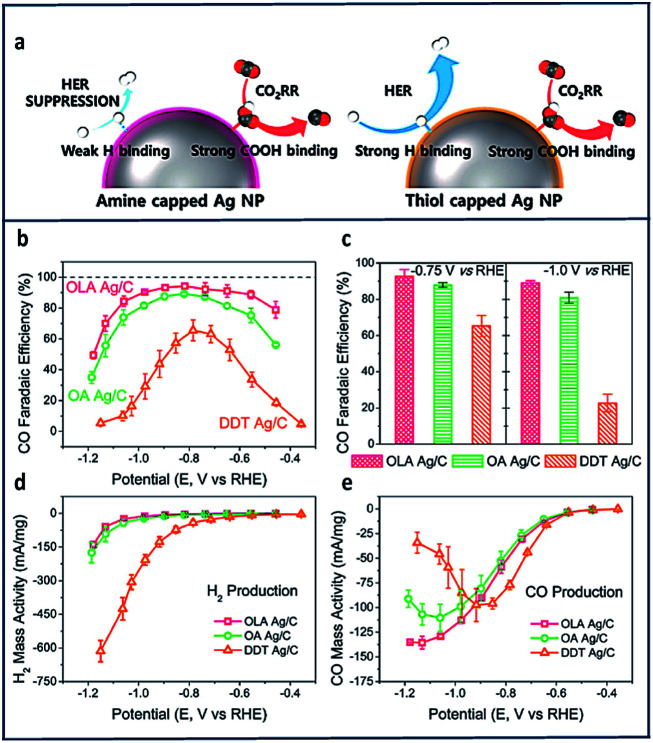
(a) Schematic of CO_2_RR on amine and thiol-capped Ag nanoparticles. Variation of FE_CO_ of OLA, OA and DDT Ag/C with (b) the applied voltage and (c) fixed potential. Mass activity for (d) H_2_ and (e) CO production of OLA, OA and DDT Ag/C at varied applied potentials.^100^ Copyright (2017) American Chemical Society.

Comparing the mass activities of the CO and H_2_ products in Fig. 10d and e, HER suppression was observed at more negative potentials (lower than −0.9 V *vs.* RHE). DDT showed the highest CO partial mass activity compared to both OLA and the oleic acid (OA) at −0.4 V to −0.9 V *vs.* RHE (Fig. 10e). They also compared the immobilization of ethylenediamine (EDA) to cysteamine onto Ag nanoparticles and found that EDA showed a higher selectivity toward CO production due to the presence of the additional amine group.

Carbon-based materials such as CNTs have proven to be a promising conductive solid support for heterogenization of molecular catalysts toward electrochemical CO_2_RR. This is due to their ability to form a strong noncovalent π–π interactions with aromatic ligands such as pyrene^150^ and porphyrin.^41,151^ Hu *et al.*^152^ reported reduction of CO_2_ to CO with an efficiency of over 90% using immobilized cobalt-tetraphenylporphyrins (CoTPPs) onto CNT in aqueous solution. Likewise, previous work by our group demonstrates selective reduction of CO_2_ to CO with a FE of 90% upon immobilization of iron–porphyrin-dimers onto CNTs.^56^ This proved to be twice as efficient as when the same catalyst was applied in a homogenous medium.

**Fig. 11:**
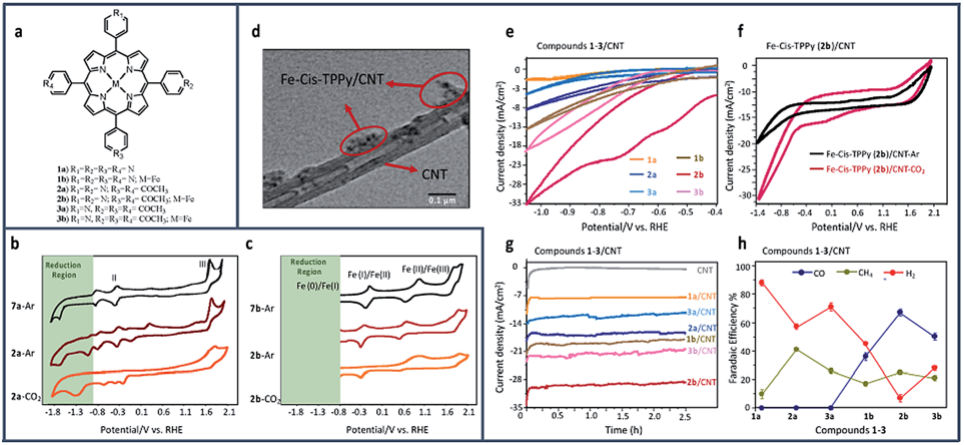
(a) Molecular structure pyridine–porphyrin complexes (b) CV comparison of 0.01 mM of TPP (7a)/GCE under argon, and *cis*-TPPy (2a)/GCE under argon and CO_2_; (c) Fe–TPP (7b)/GCE under argon, and Fe–*cis*–TPPy (2b)/GCE under argon and CO_2_ in 0.1 M NBu_4_PF_6_/DMF solution. Conditions: scan rate: 100 mV s^−1^; working electrode: glassy carbon; reference electrode: Ag/AgCl; counter electrode: platinum; (d) Transmission electtron microscopy (TEM) image of the porphyrin 2b/CNT with scale bar of 0.1 μm. CV comparison of (e) all compounds 1-3/CNT under CO_2_; (f) compound 2b/CNT in the presence and absence of CO_2_; (g) chronoamperometry comparison of 1–3/CNT at −0.6 V *vs.* RHE; (h) FE comparison of all 1–3/CNT compounds at −0.6 V *vs.* RHE in 0.1 M aqueous NaHCO_3_.^17^ Copyright (2020) American Chemical Society.

Similar enhancements to the reduction of CO_2_ to methane (CH_4_) and CO with both metalled and non-metallated iron–porphyrin–pyridine (Fe–TPPy) catalysts were seen when non-covalently immobilized onto CNTs.^17^ Among the synthesized catalysts shown in Fig. 11a, Fe-*cis* (2b)–pyridine–porphyrin catalysts, exhibited the highest current density (1.32 mA cm^−2^) and FE (76%) in reducing CO_2_ to CH_4_ and CO. Current density and product selectivity were remarkably enhanced to 30 mA cm^−2^ with the total FE of 92% after immobilization onto CNTs, comparable or higher than that of similarly reported catalysts.

Comparing the CV of non-metallated 2a/GCE in Fig. 11b under argon and CO_2_, an enhancement to the current density can be seen in the CO_2_ saturated solution stating at ∼−0.8 V *vs.* RHE. This increase in current density seen after purging 2a/GCE with CO_2_ demonstrates the important role of pyridine in the capture and electroreduction of CO_2_ to methane. Metallated isomers increased the number of available capture sites and led to a direct increase in current density for all studied compounds (Fig. 11c). As seen in Fig. 11c, the broad CO_2_ reduction peak at ∼−1.3 V *vs.* RHE aligns with the potential range observed for iron-cantered porphyrins.

**Fig. 12:**
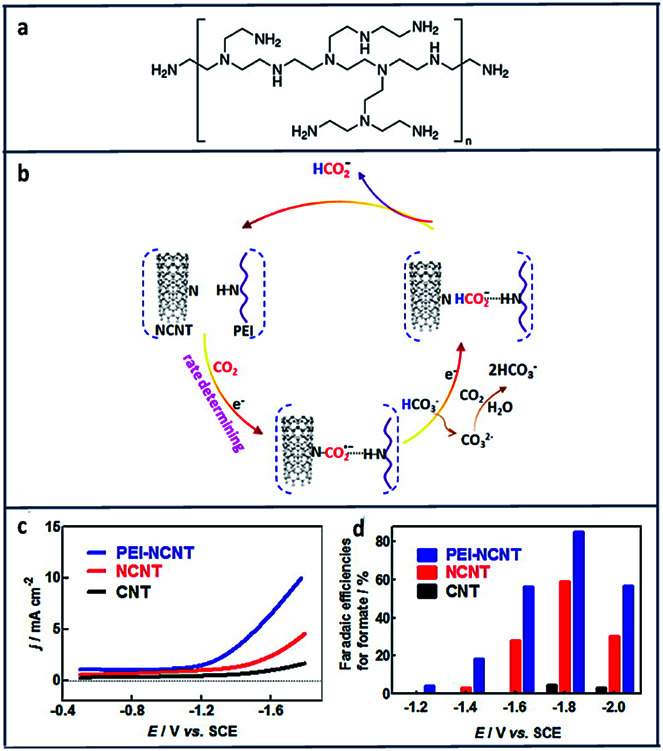
(a) Structure of branched polyethylenimine (PEI). (b) Proposed mechanism for CO_2_ reduction at PEI Functionalized, nitrogen-doped carbon nanomaterials. (c) cathodic linear sweep voltammetry scans at 50 mV s^−1^ in a CO_2_-saturated aqueous 0.1 M KHCO_3_ solution. (d) Plot of faradaic efficiencies for formate production *vs.* applied potential at CNT/GC, NCNT/GC, and PEI–NCNT/GC electrodes.^153^ Copyright (2014) American Chemical Society.

Another report suggests using polyethylenimine (PEI) (Fig. 12a) will stabilize the electroreduction of CO_2_ to HCOO^−^ through hydrogen bonding interactions (Fig. 12b).^153^ As shown in Fig. 12c and d, PEI-NCNT had the highest current density (9 mA cm^−2^) compared to nitrogen doped carbon nanotubes (NCNT) and bare CNT with a high FE of 87%.

### 4.3. Enhanced heterogeneous amine molecular catalysts using flow cells

**Fig. 13:**
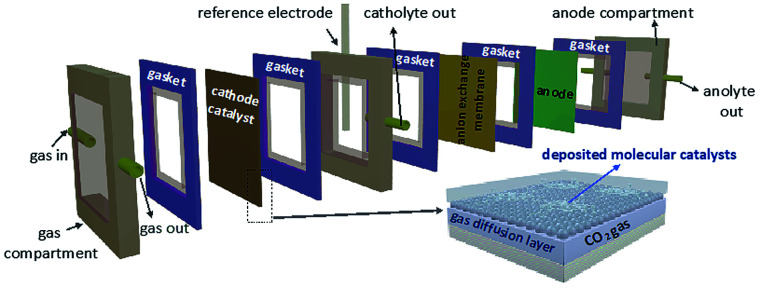
Schematic of a flow cell.^161^ Copyright (2020) American Chemical Society.

In addition to the aforementioned solid supports and immobilization techniques for heterogeneous molecular catalysis, use of flow cell electrolyzers is another technique that has been proven to enhance overall catalytic performance. This emerging system minimizes the distance between the electrode and the catalytic layer; combining efficient electrode-to-catalyst electron transfers with a continuous, single-pass directional CO_2_ delivery. These optimizations ultimately result in high energy efficiencies, product selectivities, and a reduction to operational costs.^154–160^ An additional benefit of flow cell electrolyzers is the translatability of their results to modern industrial practices. Generalized flow cell setups include a gas diffusion layer (GDL) which is directly exposed to the electrolyte solution (Fig. 13).^161–163^ The catalyst layer is typically deposited directly onto the GDL, allowing for a greater effective catalyst surface area.

Recent studies of molecular catalysts operated in flow cells find significant gains to both product selectivity and reaction conversion rate. Cobalt and iron porphyrin and phthalocyanine complexes deposited onto a gas diffusion electrode through non-covalent bonding in a flow cell have been reported to achieve high current densities and selectivities.^164,165^ An example of immobilized cobalt and iron amino molecular catalysts on carbon paper supports report current densities up to 165 mA cm^−2^ while maintaining high product selectivity (up to 94%).^161,165^ These results, confirm the importance of state-of-the-art noble molecular based catalysts for electrochemical CO_2_RR.

## 5. Conclusions and future prospects

A wide range of amine-based molecular catalysts has been explored for the electrochemical reduction of CO_2_ over the years and the contributions of small molecule catalysis to finding insights into the mechanism of electrochemical CO_2_RR is instrumental to the intelligent design of new catalysts. In particular, the role of amine-based ligands and functional groups were found to play an important role in capturing CO_2_ itself and being used as covalent linkers for direct immobilization.

Although insights into the intricacies of CO_2_RR have been garnered thanks to thorough studies of immobilization techniques, the influence of metal electrodes, and the role of different metal centers in organometallic compounds, further improvements to catalytic activity and stability are still needed before large-scale application can be realized. As described in this review, noncovalent and covalent immobilization can be achieved through various techniques to positive effect. Expanding on this new approach, many renewed studies on both homogeneous and heterogeneous systems are gaining greater traction with promising bounds being made every year.

Various strategies can be considered to overcome the current limitations in the electrochemical reduction process for CO_2_ using amine-based molecular catalysts. For homogeneous electrocatalysis; (i) synthesizing small amine molecules that have a high affinity towards CO_2_ but have a weaker amine–CO_2_ bond; (ii) developing new nanostructured catalysts with large electrochemically active surface areas to facilitate the reduction process of the amine–CO_2_ at lower potentials and high catalytic activity and selectivity would be promising next steps. For heterogeneous systems: (i) developing facile synthetic approaches to amine-functionalized MOFs; (ii) preparing high amine content MOFs with improved chemical stability; and (iii) improving immobilization strategies with nanostructured materials instead of the smooth metal surfaces are recommended to achieve higher catalytic performance.

Therefore, further investigations are required to achieve high stability and catalytic activity of the amino electrocatalysts to understand the fundamental kinetics of CO_2_ reduction, and the effectiveness of the catalysts.

## Conflicts of interest

There are no conflicts to declare.

## Supplementary Material
